# The necessity of booster vaccination after neonatal hepatitis B vaccination

**Published:** 2011-06-01

**Authors:** Kamran Bagheri Lankarani

**Affiliations:** 1 Health Policy Research Center, Shiraz University of Medical Sciences, Shiraz, IR Iran

**Keywords:** Booster immunization, Vaccination, Hepatitis B vaccine

Soon after introducing recombinant hepatitis B virus (HBV) vaccine, universal neonatal vaccination became the corner stone of the preventive measures for this potentially life threatening infection[1][2][3]. By 2006 more than 177 out of 193 member states of world health organization (WHO) introduced HBV vaccination in their national infant immunization programs[1]. Following a complete series of vaccination during neonatal period, protective antibody level raises in more than 95% of infant's blood test up to 18 months after vaccination [3][4][5]. The effectiveness of this strategy has been shown by several investigators in reducing the incidence of HBV carrier rate and probably cirrhosis and hepatocellular carcinoma [1][2][5][6][7].

A level of 10 IU/L of anti hepatitis B surface antibody (Anti HBsAb) is usually considered as a protective level against future infections. Although this level was initially determined in studies about passive prophylaxis of HBV infection, the same level was arbitrary applied to active immunization though this was debated by some researchers [8][9][10]. Universally there is a consistent decline in antibody titer over time .The reported rate of persistent protective level of Anti HBsAb titers varied from 33% up to 79%, at least 5 year after vaccination [10][11][12][13][14]. Currently about 21% of worldwide HBV related mortalities are linked to the vertical transmission, and the other depends on unsafe injection and high risk behavior that causes more concern of long lasting immunity extending to adolescent and even adulthood[1][2][5].

Tosun et al. [15] in a published article in Hepatitis Monthly reported that the protective level of antibodies 9 years after neonatal vaccination was roughly near 50% in children from Turkey [15]. Although the authors of the mentioned study have checked the health records and enrolled only those received complete vaccination, there were still many infants who did not receive vaccination appropriately. So, there was a wide variation of utilization of HBV vaccination both within and between countries.

The coverage of complete vaccination of infants against HBV was estimated 69% in 2008 worldwide[1][5] while this rate for neonatal received vaccination within 24 hours after birth was about 27% globally with a wide variation among countries (3-71%)[1][5]. Later immunization may leave the neonates vulnerable to possible transmissions both horizontally (e.g. within the health services or through household contacts with infected family members) and vertically [2][8][9] that leads to WHO recommendation regarding implementation of neonatal vaccination against HBV within the first 24 hours of life [5][9].

Some problems such as maintaining cold chain in transportation and handling of vaccine, improper injection and other technical problems in this context are still real challenges in many countries making effectiveness of vaccination, like HBV which need a stable cold chain, lower than expected in real practice [2][5][8][9]. For instance a recent study from Mongolia mentioned that the HBV incidence rate after 7-12 years of vaccination was more than two times in rural infants who received HBV vaccination in winter compared to other seasons [16] which probably is related to vulnerable cold chain. Some studies showed that those who were born of infected mothers specially mothers with higher HBV DNA level have lower rate of vaccination response [17][18]. According to available studies on HBV screening during pregnancy in Turkey, 2.1-12.3% of Turkish women infected with HBV in different geographic locations [19][20][21]. The status would be probably much higher if Anti HBc Ab was measured. In Tosun's study only two children were HBsAg positive (0.15%). There would be a concern whether a proportion of non-responders have been born to HBV infected mothers and were infected vertically. There is also possibility of being infected horizontally in early childhood.

The HBV infection rate in children received vaccination after determining by Anti HBcAb positivity might be as high as 0.5% [11]. The impacts of childhood infection probably would not be high in the population studied by Tosun although in her study Anti HBcAb status of children were not evaluated. Tosun et al. also reported lower protection rate in lower socioeconomic groups compared to middle and high classes and in those who were delivered in health institution versus those who were delivered elsewhere. There are many reports on high prevalence of HBV carriers [1][2][5][9]and few HBV vaccination [9][22][23] among lower socioeconomic groups. But we are not informed of any previous reports showing the lower response rate to HBV vaccine in lower socioeconomic classes. There could be many confounding factors including malnutrition, concomitant diseases or even receiving vaccine in bad techniques such as inappropriate handling of vaccine including stable and sustained the cold chain [8][9][16][24][25]. This issue needs to be confirmed in larger studies and needs to be analyzed in more details to find the possible etiology of such difference.

After all we still confront with this major question: How long the protective effect of neonatal HBV vaccination persists? Is there a need for booster after complete series of vaccination? If so when this booster should be administered and to whom? Should it be universal like the neonatal vaccination itself or only be given after monitoring or given only to selected groups?

Answer to these questions is not easy and there are contradictory evidences in the literature. The debate is whether the reduction in antibody levels is indicating lower immunity or not. An anamnestic response may occur when people who were previously immunized against an antigen are re-exposed to the same antigen [9][10][26]. How can we be sure that the same would not be happened to the children when they re-exposed to the virus? The persistence of specific B cell population committed to antibody production against HBsAg has been shown years after vaccination [26]. In a recent report from Taiwan, researchers have shown that after 20 years post vaccination the immune system memory is lost in up to 80% of vaccines [27]. As with all experimental studies it would be debatable to apply this laboratory based study to real practice specially considering other contradictory studies [9][10][11][12][28][29]. A recent Cochrane review was not able to identify any randomized study to assess the benefit of booster vaccination in prevention of HBV [30].

The same group reported the rate of breakthrough infection among vaccines was less than 0.1% [31]. Even chronic infection may not develop in those patients if they have completed their HBV vaccine series. They also concluded the protection provided by complete series of monovalent HB vaccine persists for at least two decades in the vast majority of general population and they found that there was no evidence of need for booster vaccination, although they recommended further studies to assess the need for booster in different subgroups [31]. In conclusion we still do not have enough evidence to recommend universal booster vaccination. None of the international guidelines for HBV vaccination currently recommend booster dose [1][2][3][5][8][9]. Probably in some high risk groups this might be of real concern though evidence for this recommendation does not exist. We need more detailed long term studies to change our current recommendation of no need for booster vaccination.
